# Plasma exchange therapy for severe gastrointestinal involvement of Henoch Schonlein purpura in children

**DOI:** 10.1186/1546-0096-12-S1-P357

**Published:** 2014-09-17

**Authors:** Ozge Basaran, Banu Celikel Acar, Nermin Uncu, Fatma Semsa Caycı, Abdurrahman Kara, Gokce Gur, Aysel Taktak, Nilgun Cakar

**Affiliations:** 1 Pediatric Nephrology and Rheumatology, Ankara Child Health Hematology Oncology Education and Research Hospital, Ankara, Turkey; 2 Pediatric Hematology, Ankara Child Health Hematology Oncology Education and Research Hospital, Ankara, Turkey

## Introduction

Henoch-schönlein purpura (HSP), which is predominantly a disease of childhood, is a small vessel vasculitis. It is characterized by non-thrombocytopenic purpura, arthritis and arthralgia, abdominal pain, gastrointestinal hemorrhage and glomerulonephritis. Prognosis and treatment opportunities depends on the clinical severity and organ involvement. Some reports have been published suggesting the beneficial effects of plasma exchange in HSP nephritis, but there have been only some small case series discussing the efficacy of plasma exchange in gastrointestinal system (GIS) involvement.

## Objectives

The aim of this report is to evaluate the plasma exchange as a choice for the treatment of life threating GIS ınvolvement in HSP when refractory to conventional therapies.

## Methods

We respectively reviewed the medical records of HSP patients whom had plasma exchange therapy due to massive GIS involvement. We reported age, gender, initial HSP presentation, etiological or triggering factors and disease course. Treatment modalities, side effects and their outcomes were noted.

## Results

Seven patients who had plasma exchange therapy due to GIS involvement were identified. All patients had pulse methylprednisolone (MPZ) treatment and then continued with oral prednisolone (2mg/kg/day) therapy. All patients’ complaints continued, GI bleeding and the severity of disease did not improve. Therefore, pulse cyclophosphamide was added to the treatment. Two patients received intravenous immunoglobulin (IVIG) therapy. Gastrointestinal manifestations did not improved and plasma exchange was performed. All patients improved after plasma exchange management.

## Conclusion

Treatment of GI involvement in HSP with plasma exchange have been mainly based on case reports. According to our data, we propose that, plasma exchange may be a safe and efficient management choice in pediatric HSP patients with massive GIS involvement

## Disclosure of interest

None declared

